# Frailty and incident heart failure in older men: the British Regional Heart Study

**DOI:** 10.1136/openhrt-2021-001571

**Published:** 2021-06-04

**Authors:** Douglas GJ McKechnie, A Olia Papacosta, Lucy T Lennon, Sheena E Ramsay, Peter H Whincup, S Goya Wannamethee

**Affiliations:** 1 Department of Primary Care and Population Health, University College London, London, UK; 2 Population Health Sciences Institute, Newcastle University, Newcastle upon Tyne, Newcastle upon Tyne, UK; 3 Population Health Research Institute, St George's University of London, London, UK

**Keywords:** heart failure, epidemiology, risk factors

## Abstract

**Objective:**

Frailty and heart failure (HF) are cross-sectionally associated. Published longitudinal data are very limited. We sought to investigate associations between frailty and incident HF.

**Methods:**

Prospective study of 1722 men, examined at age 72–91 years. Scores based on the Fried phenotype, Gill index and a novel frailty score, based on the Health Ageing and Body Composition Battery, incorporating slow walking speed, low chair-stand time and subjective difficulty with balance, were calculated. Associations between these scores and incident HF were analysed with Cox proportional hazard modelling.

**Results:**

1445 men with frailty data and without prevalent HF were included. 99 developed HF (mean follow-up 6.1 years). Men scoring 3/3 on our novel frailty score had elevated risk of incident HF (HR 2.77, 95% CI 1.25 to 6.15), which persisted after adjustment for established risk factors and interleukin-6 (HR 3.14, 95% CI 1.35 to 7.31). This risk remained increased, although attenuated, after excluding HF events within 2 years of baseline (HR 2.05, 95% CI 0.61 to 6.92). The frailty phenotype showed a non-significant association with HF (age-adjusted HR 1.92, 95% CI 0.99 to 3.73), which was further attenuated after adjustment for prevalent coronary heart disease and Body mass index (HR 1.60, 95% CI 0.81 to 3.15). Gill-type scores were weakly associated with HF risk after these adjustments (HR 1.31, 95% CI 0.47 to 3.70).

**Conclusion:**

In these older men, the combination of slow walk speed, low sit-stand time and balance problems were associated with high risk of incident HF, independent of established risk factors and inflammatory markers. However, undiagnosed HF at baseline may still be a confounder. There is a differential association between aspects of the frailty phenotype and incident HF.

**Key questions**

**What is already known about this subject?**

- Heart failure (HF) and frailty are associated in cross-sectional studies, and probably share common pathophysiological determinants. Longitudinal data are very limited; only one published study has reported an association between frailty and the development of HF.

**What does this study add?**

- Frailty, as measured by the combination of slow gait speed, low chair-stand time and difficulty with balance, is associated with elevated risk of developing HF, even after adjusting for comorbidities, established risk factors and inflammation. Frailty as defined by the Fried frailty index showed a weaker association with HF risk. We found no associations between a score based on the Gill index and HF.

**How might this impact on clinical practice?**

- People who are frail should be considered at higher risk of developing HF. Further work might lead to frailty assessment, based on the criteria we describe here, being used as part of HF risk prediction scores. Interventions to prevent or ameliorate frailty might help to reduce the subsequent development of HF.

## Introduction

Frailty, from the Latin fragilis (brittle), describes multisystemic physiological dysfunction conferring vulnerability to relatively minor health stressors. Frailty is strongly associated with older age and often co-occurs with multimorbidity.^1^

Heart failure (HF) and frailty frequently coexist.^2^ People with HF who are frail experience higher mortality rates, higher hospitalisation rates and greater disability.^3^ The mechanistic features of HF and frailty overlap, with ageing, chronic inflammation, comorbidity and sarcopenia implicated in both, and when the two coexist, there seems to be a bidirectional relationship, with each worsening the effects of one other.^4^

Frailty and HF have been examined in many cross-sectional studies, but there is a paucity of longitudinal data, with a recent systematic review identifying only two longitudinal analyses of frailty and HF.^5^ In the Women’s Health Initiative Observational Study, a large cohort of women aged 65–79, the presence of HF showed no association with frailty risk at 3-year follow-up.^6^ Conversely, in the Health Ageing and Body Composition (HABC) Study cohort, among participants aged 70–79, frailty at baseline in people was associated with increased risk of incident HF.^7^ This risk persisted despite adjustment for the HABC Risk score (based on age, heart rate, smoking status, fasting glucose, creatinine, systolic blood pressure and serum albumin), inflammatory markers, ankle arm index, incident coronary heart disease and competing mortality. Two measures of frailty were used: the HABC battery, a composite of gait speed, five serial chair stands, narrow walk time and standing balance^8^; and the Gill index, based on gait speed and chair stand tests.^9^

We have previously demonstrated cross-sectional associations between cardiovascular risk factors and a frailty score based on the commonly used Fried frailty phenotype,^10 11^ which integrates exhaustion, unintentional weight loss, low physical activity, slow walking speed and low grip strength.

We; therefore, sought to determine whether frailty as measured by three different measures of frailty (based on Fried, Gill and HABC) is prospectively associated with HF risk in older men, allowing for potential confounders.

## Methods

### British Regional Heart Study

The British Regional Heart Study is a prospective study of 7735 men, aged 40–59 at enrolment, drawn from one general practice in each of 24 British towns. Sampling aimed to reflect the socioeconomic structure of those towns. Over 99% of the participants were of White European ethnicity.

Initial screening took place from 1978 to 1980. This paper uses data from the 30-year examination, when men were aged 72–91 years. All surviving men in 2010–12 (n=3137) were invited to attend a reassessment which included a questionnaire, physical examination, ECG and provision of a fasting blood sample.

### Questionnaire data

All participants completed a questionnaire regarding their lifestyle, medical and medication history. Tobacco usage was categorised as: never-smokers, long-term ex-smokers (stopped smoking ≥10 years prior), recent ex-smokers (stopped smoking <10 years prior) and current smokers. Heavy alcohol use was defined as average consumption of ≥42 UK units (1 unit=10 g) of alcohol per week. Items included self-report of walking pace (slow/steady average/fast), difficulty in keeping balance (yes/no), change in weight (decreased/increased/both increased and decreased/not changed/don’t know) and unintentional weight loss.

### Prevalent comorbidities

Prevalent HF, stroke (ischaemic or haemorrhagic) and myocardial infarction (MI) were defined as a confirmed doctor’s diagnosis prior to the baseline examination, based on primary care records. Prevalent diabetes mellitus was defined as either a physician-confirmed diagnosis of diabetes mellitus, or a fasting serum glucose of greater than 7 mmol/L.

### Physical examination

Blood pressure was measured with an Omron sphygmomanometer twice in the right arm, with the subject seated and the arm supported. The mean of the two readings was used for analysis. With subjects in light clothing and without shoes, height was measured with a Harpenden stadiometer to the last complete 0.1 cm, and weight with a Tanita MA-418-BC body composition analyser (Tanita, Tokyo, Japan). Body mass index (BMI) was calculated as weight/(height)^2^ (kg/m^2^). Grip strength (in kilograms) was measured using a Jamar Hydraulic Hand Dynamometer, with three measurements taken with each hand and the best of six used for the analysis. A walking test measured the time taken, in seconds, to walk 3 m at normal walking pace. A five-repetition sit-to-stand test was performed: participants were asked to move from a seated to a standing position five times in succession, avoiding the aid of hands and the time taken, in seconds, to perform this was recorded. Forced expiratory volume in 1 s (FEV1) was measured using a Vitalograph Compact II instrument with subjects standing, without nose clips. FEV1 was standardised to the average study height, 1.71 m, using the formula: standardised FEV1=FEV1 × (1.71/height)^2^.

### Electrocardiography

A twelve-lead resting ECG was recorded using a Siemens Sicard 460 instrument and classified using the Minnesota Coding scheme.^12^ ECG left ventricular hypertrophy (LVH) was defined as the presence of either Minnesota codes 3.1 or 3.3 (high amplitude R waves) plus evidence of left ventricular strain, defined as the presence of codes 4.1 or 4.2 (ST depression) or codes 5.1 or 5.2 (T wave inversion). The presence of Q waves was defined by Minnesota codes 1.1, 1.2 and 1.3 (and subcodes) in any of the anterolateral, anterior or inferior/posterior sites. Atrial fibrillation (AF) was defined as Minnesota codes 8.3.1 and 8.3.3.

### Biomarker measurement

Fasting serum samples were obtained. Glucose was measured in a fluoride oxidase plasma sample and creatinine measured using enzymatic colorimetric assays. Estimated glomerular filtration rate (eGFR) was calculated using the Modification of Diet in Renal Disease (MDRD) equation.^13^ Plasma interleukin-6 (IL-6) was measured using ELISA (R&D systems, Oxon, UK). N-terminal pro-B-type natriuretic peptide (NT-pro-BNP) was measured by electrochemiluminescence immunoassay (Roche Diagnostics, Burgess Hill, UK).

### Frailty scoring

Three different frailty scores were calculated for each participant. A score based on the Fried frailty phenotype^11^ was calculated from five variables: unintentional weight loss (a decrease in self-reported weight that respondents felt was unintentional); exhaustion (answering ‘no’ to the question ‘Do you feel full of energy?’); weakness (lowest fifth of grip strength distribution); low physical activity (self-report of being less active or much less active than an average man); and slow walking speed (lowest fifth of walking speed). Where measured walking speed was unavailable, self-report of low walking pace was used (self-report of walking speed, or being unable to walk more than a few steps, or <200 yards (approximately 180 m), or difficulty walking across a room), Participants with none of these features were defined as ‘robust’; with one or two as ‘pre-frail’; and with three or more as ‘frail’.

To aid comparisons to prior work, we calculated frailty scores analogous to the Gill index and HABC battery. The Gill index measures speed over 6 m (<0.6 m/s^2^=1 point) and ability to stand from a seated position with arms folded (unable=1 point). Our ‘Gill-like’ score assigned one point for walk speed <0.6 m/s^2^ over 3 m, and one point for inability to perform five chair-stands without use of hands (or inability to perform at all). A total score of 1 corresponds to ‘moderate’ frailty and 2 to ‘severe’ frailty.^9^

The HABC battery is a continuous composite score of performance on four tests: measured gait speed over 6 m, five serial chair stands, narrow walk over 6 m and timed standing balance over 30 s.^8^ Since we did not have direct measurements of narrow walk or balance time, we could not compute a continuous ratio score. We constructed a new interval score measure of frailty based on three variables: 3 m walk speed (1 point if <0.6 m/s, or if unable to do at all), time to perform five serial chair stands without use of hands (1 point if ≥18 s, if unable to do without hands or at all) and self-reported difficulty maintaining balance (1 point if answering ‘yes’ to the question ‘Do you currently have difficulty carrying out any of the following activities on your own as a result of a long term health problem—Keeping your balance?’). Cut-offs for walk time and sit-stand time were derived by visual inspection of scatter plots, showing inflection points at approximately 0.6 m/s and 18 s respectively. This is henceforth referred to as the ‘novel’ score.

### Exclusion criteria

Men with a diagnosis of HF at baseline were excluded from analysis, as were men without data on any of the three frailty scores. Analyses were handled using complete-case analysis at that step of the analysis: participants with missing values for any variable within that analytic step were excluded.

### Follow-up

All men were followed up to June 2018 for cardiovascular morbidity and mortality. Mortality was obtained via the National Health Service Register. Incident HF was defined as a confirmed doctor’s diagnosis of HF from primary care records, and verified, where possible, using clinical information from primary and secondary care records, as well as from death certificates with International Classification of Diseases (ICD)-9 code 428. Time to incident HF was the time between the baseline examination and confirmed doctor’s diagnosis of HF.

### Statistical analysis

All statistical analyses were performed using SAS software, V.9.4 of the SAS System for Windows.

Descriptive statistics were used to report sample characteristics at baseline, with χ^2^ tests used for comparisons between groups for categorical variables. The t-tests were used for comparisons of normally distributed variables. Distributions for NT-pro-BNP and IL-6 were positively skewed. Geometric means were calculated for these variables and comparisons made using the Kruskal-Wallis test. These variables were natural log-transformed for further analysis.

Cox proportional hazard modelling was used to assess associations between frailty score group and the relative risk of incident HF over time, correcting for potential confounders. Non-frail groups were used as the reference.

Four stepwise analyses were performed to assess how associations between incident HF and frailty score attenuated with progressive adjustment. The initial model included frailty score and age as independent variables. The second model added prevalent MI and BMI. The third model added other known risk factors for HF: systolic blood pressure; diabetes mellitus; use of blood pressure lowering medication; FEV1; smoking status; eGFR; AF; Q waves; and LVH on ECG, plus prevalent stroke at baseline, as a cardiovascular disease associated with frailty. The fourth model added log-transformed IL-6 as an independent variable. IL-6, a proinflammatory cytokine, has been associated with incident HF risk.^14 15^

These analyses were repeated with the Fried scoring system, the Gill-like scoring system and the novel frailty score.

To demonstrate trends across groups, these analyses were repeated with the frailty groups assigned integer scores: 0–2 for the Fried (0=robust, 1=pre-frail, 2=frail) and Gill (0=non or mildly-frail, 1=moderately frail, 2=severely frail) phenotypes and 0–3 for the novel score phenotypes. These were then modelled continuously as independent variables.

The proportional hazards assumption was tested by including interactions of the predictors and a function of survival time into the models. None of the time-dependent covariates were statistically significant, suggesting the proportional hazards assumption was not violated.

### Supplementary analyses

Three further supplementary analyses were conducted: first, to examine the effect of early HF diagnoses during follow-up (which may indicate asymptomatic, or symptomatic, but un-diagnosed, HF), the analyses above were repeated, but excluding men who developed HF within 2 years of baseline.

Second, we added baseline log NT-proBNP as an independent variable to the fully adjusted main analysis. NT-pro-BNP is elevated in HF, and also a potent predictor of incident HF^16^; adjusting for NT-pro-BNP levels at baseline may, therefore, partially adjust for undiagnosed HF as a confounder.

Thirdl to determine the effect of our approach to missing values, we repeated the four stepwise analyses, restricted only to cases with complete data for the final (fully adjusted) model.

## Results

**Figure 1:**
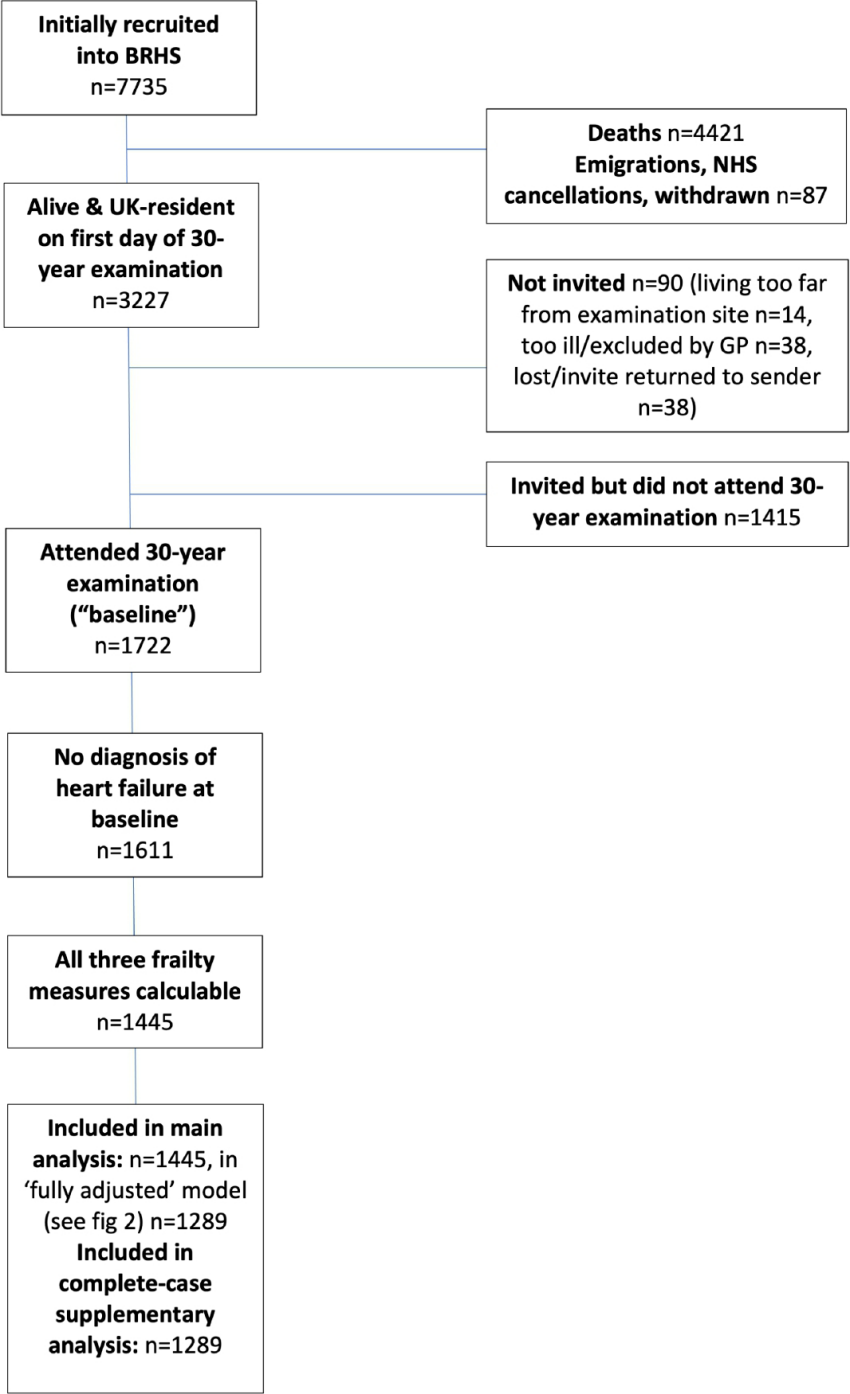
Participant flow diagram. BRHS, British Regional Heart Study; GP, general practitioner; NHS, National health Service.

A total of 1722 men (55% of the surviving cohort) attended the baseline examination. A total of 111 men with prevalent HF and 166 men missing at least one frailty score were excluded, leaving 1445 men for analysis. A participant flow diagram is given in figure 1.

### Baseline characteristics of participants

Table 1 gives baseline characteristics of participants. In bivariate analyses, mean age and NT-pro-BNP were significantly higher in the group that developed incident HF versus those who did not; mean FEV1 was significantly lower in those who developed incident HF. Men who developed incident HF were more likely to have had a prior MI, a prior stroke, to have Q waves on baseline ECG, and to have AF than those who did not. There were significant differences in the proportions of men in the different Fried phenotype (p*=*0.047) and novel frailty score (p=0.013) groups between those with and without incident HF, but not in the proportions of those in the different Gill score groups (p=0.32).

**Table 1:** Characteristics of study participants at baseline

	Did not develop HF (n=1346)	Developed HF (n=99)	P value
Age (years)	78.1 (4.5)	79.8 (4.9)	0.0004
Smoking status			0.42
Never smoked	560 (38.6%)	34 (34.3%)	
Long-term ex-smoker	720 (53.5%)	55 (55.6%)	
Recent ex-smoker	62 (4.6%)	4 (4.0%)	
Current smoker	43 (3.2%)	6 (6.1%)	
Prior myocardial infarction	158 (11.7%) Missing=0	22 (22.2%) Missing=0	0.002
Prior stroke	78 (5.8%) Missing=0	12 (12.1%) Missing=0	0.01
Diabetes mellitus	214 (16.0%) Missing=5	15 (15.2%) Missing=0	0.83
Taking antihypertensive medication	707 (52.5%) Missing=0	57 (57.6%) Missing=0	0.33
Physical measurements			
Body mass index (kg/m^2^)	27.0 (3.8) Missing=11	27.7 (3.8) Missing=1	0.13
Systolic blood pressure (mm Hg)	146 (18.8) Missing=1	144 (18.9) Missing=0	0.38
Diastolic blood pressure (mm Hg)	76.6 (11.4) Missing=1	75.9 (11.4) Missing=0	0.61
Forced expiratory volume in 1 s (L)	2.46 (0.56) Missing=35	2.24 (0.56) Missing=2	0.0002
Electrocardiographic measurements			
Left ventricular hypertrophy	99 (7.4%) Missing=9	9 (8.1%) Missing=0	0.80
Atrial fibrillation	93 (7.0%) Missing=9	16 (16.2%) Missing=0	0.0008
Q waves present	75 (16.5%) Missing=9	24 (24.2%) Missing=0	0.047
Biomarkers			
Estimated glomerular filtration rate	74.0 (17.5) Missing=62	71.7 (19.1) Missing=4	0.23
Interleukin-6	2.94 (1.76–4.34) Missing=77	3.37 (2.10–4.92) Missing=4	0.06
NT-pro-BNP	121 (64.0–250) Missing=89	317 (133–746) Missing=6	<0.0001
Frailty scores			
Fried phenotype			0.047
Non-frail (0)	400 (29.7%)	18 (18.2%)	
Prefrail (1)	740 (55.0%)	62 (62.6%)	
Frail (2)	206 (15.3%)	19 (19.2%)	
Gill-like score			0.32
Mild or no frailty (0)	1212 (90.0%)	85 (85.9%)	
Moderate frailty (1)	97 (7.2%)	9 (9.1%)	
Severe frailty (2)	37 (2.8%)	5 (5.1%)	
Novel frailty score			0.013
0	967 (71.8%)	57 (57.6%)	
1	260 (19.3%)	26 (26.3%)	
2	76 (5.7%)	9 (9.1%)	
3	43 (3.2%)	7 (7.1%)	

For normally distributed continuous variables, values are mean (SD) and p values for t tests; for NT-pro-BNP and interleukin-6, values are geometric mean (IQR) and p values for Kruskall-Wallis tests; for categorical variables values are N (% of total by column) and p values for χ2 tests.

HF, heart failure; NT-proBNP, N-terminal pro B-type natriuretic peptide.

### Incident HF risk

Mean follow-up time was 6.1 years (SD 1.8 years). Table 2 shows the rate of incident HF (per 1000 person-years) per frailty score group.

**Table 2:** Incident rates of HF per 1000 person-years for participants in each frailty score group

	No of incident HF cases	Rate/1000 person-years
Fried phenotype		
Non-frail (n=418)	18	6.60
Prefrail (n=802)	62	12.1
Frail (n=225)	19	15.8
Gill-like score		
Non-frail or mildly frail (n=1297)	85	10.5
Moderate frailty (n=106)	9	16.2
Severe frailty (n=42)	5	24.4
Novel frailty score		
0 (n=1024)	57	8.82
1 (n=286)	26	15.1
2 (n=85)	9	20.6
3 (n=50)	7	29.5

HF, heart failure.

**Figure 2:**
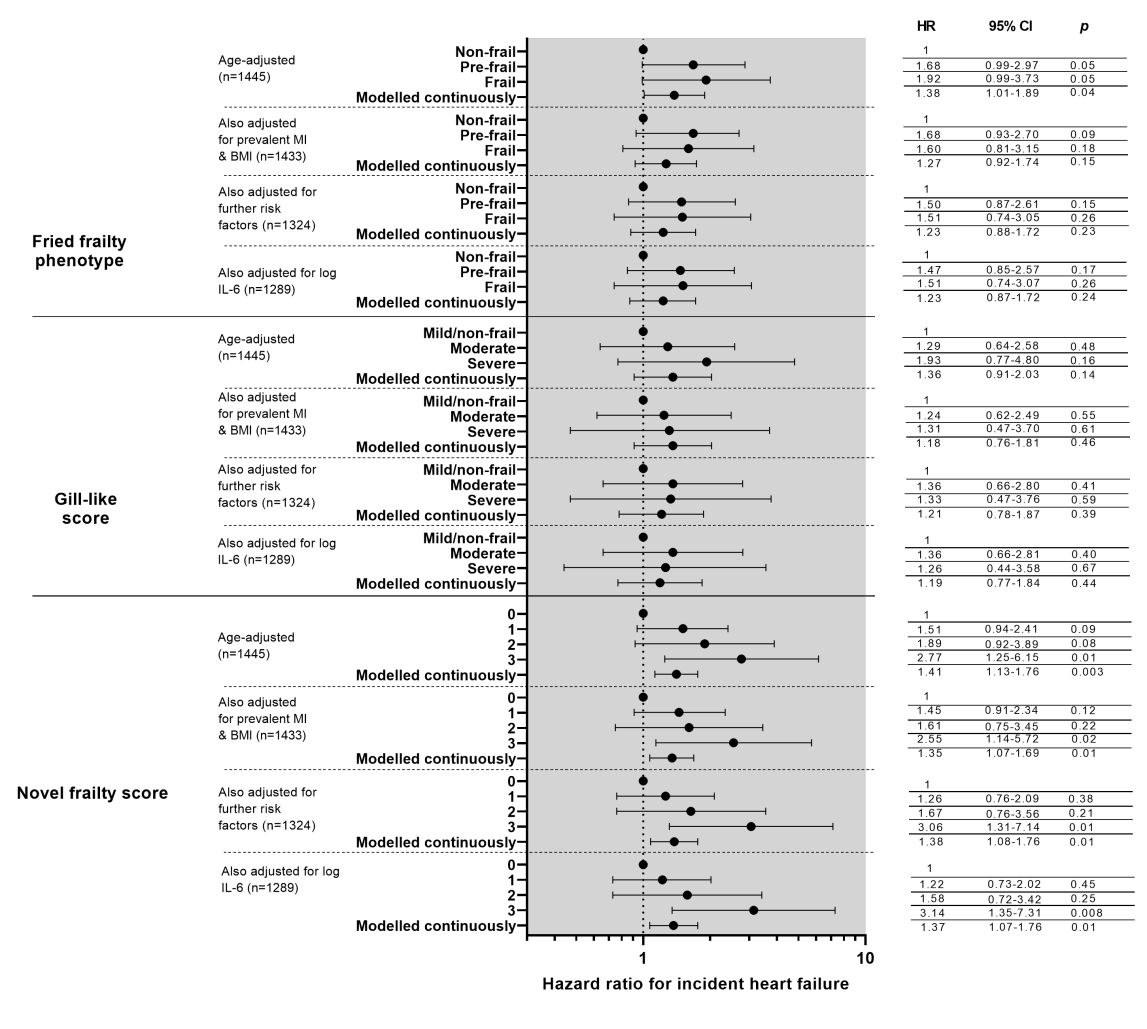
Cox proportional hazard modelling of risk of incident heart failure. ’Further risk factors’ are prevalent stroke, systolic blood pressure, smoking status, use of blood pressure lowering medications, EGFR, FEV1, atrial fibrillation, Q waves, and left ventricular hypertrophy. analyses are presented first with comparisons with the reference (robust/least frail) modelled as categorical variables, then trend across groups modelled as continuous variables. BMI, body mass index; EGFR, estimated glomerular filtration rate; FEV1, forced expiratory volume in 1 s.

Figure 2 shows the results of Cox proportional hazard modelling of incident HF risk for all three frailty scoring systems, with progressive adjustment for likely confounders.

Men who were prefrail or frail, as defined by the Fried phenotype, showed a non-statistically significant association with HF risk (HR 1.68, 95% CI 0.99 to 1.92, p=0.05 and HR 1.92, 95% CI 0.99 to 3.73, p=0.05, respectively). Adjustment for prevalent MI and BMI attenuated the association, as did further adjustment for clinical parameters. When modelled continuously for trend, there was a significant association between higher Fried frailty score and risk of incident HF in the model adjusting for age only (HR 1.38, 95% CI 1.01 to 1.89, p=0.04) but not with subsequent adjustments.

There were no significant differences in HF risk between non-frail or mildly frail men and moderately or severely frail men as defined by the Gill-like score in any of the models, nor when the scores were modelled continuously.

Men with the highest novel frail score had a higher HF risk compared with those in the lowest group in the age-adjusted model (HR 2.77, 95% CI 1.25 to 6.15, p=0.01), which persisted after adjusting for prevalent MI and BMI (HR 2.55, 95% CI 1.14 to 5.72, p=0.02), additional clinical risk factors (HR 3.06 95% CI 1.31 to 7.14, p=0.01) and after adjustment for log IL-6 (HR 3.14, 95% CI 1.35 to 7.31, p=0.008). When modelling the frailty severity score continuously for trend, there were statistically significant associations between higher novel frailty scores and incident HF risk in all analyses (HR 1.37, 95% CI 1.07 to 1.76, p=0.01 in the fully adjusted model).

### Supplementary analyses

The results of a supplementary analysis, excluding 23 participants who developed incident HF within 2 years of the baseline examination, are given in table 3—for brevity, only the age-adjusted and fully adjusted models are given. Most associations were notably weaker than in the main analyses. The association between HF risk and the top group of the novel frailty score lost significance, but those in the second-highest group had a significant positive association with HF risk (age adjusted, HR 2.70, 95% CI 1.29 to 5.62, p=0.008), which was retained after full adjustment (HR 2.24, 95% CI 1.02 to 4.95, p=0.05).

**Table 3:** Results of Cox proportional hazard modelling of risk of incident heart failure, in a subgroup analysis excluding HF events within 2 years of baseline

	HR	95% CI of HR	P value
Fried phenotype			
Adjusted for age only (n=1422)			
Prefrail vs non-frail	1.64	0.91 to 2.94	0.10
Frail vs non-frail	1.59	0.73 to 3.47	0.24
Modelled continuously for trend	1.28	0.90 to 1.84	0.17
Fully adjusted* (n=1268)			
Prefrail vs non-frail	1.44	0.78 to 2.67	0.25
Frail vs non-frail	1.15	0.49 to 2.69	0.75
Modelled continuously for trend	1.10	0.75 to 1.62	0.63
Gill-like score			
Adjusted for age only (n=1422)			
Moderate vs mild/no frailty	1.18	0.51 to 2.75	0.70
Severe vs mild/no frailty	1.68	0.52 to 5.37	0.38
Modelled continuously for trend	1.26	0.76 to 2.08	0.80
Fully adjusted* (n=1268)			
Moderate vs mild/no frailty	1.25	0.52 to 2.98	0.62
Severe vs mild/no frailty	0.92	0.22 to 3.88	0.90
Modelled continuously for trend	1.03	0.59 to 1.80	0.93
Novel frailty score			
Adjusted for age only (n=1422)			
1 vs 0	1.43	0.83 to 2.46	0.20
2 vs 0	2.70	1.29 to 5.62	0.008
3 vs 0	1.69	0.52 to 5.49	0.38
Modelled continuously for trend	1.38	1.06 to 1.79	0.02
Fully adjusted* (n=1268)			
1 vs 0	1.15	0.64 to 2.07	0.93
2 vs 0	2.24	1.02 to 4.95	0.05
3 vs 0	2.05	0.61 to 6.92	0.25
Modelled continuously for trend	1.34	1.00 to 1.80	0.05

*Fully adjusted=adjusted for age, prevalent myocardial infarction, prevalent stroke, systolic blood pressure, smoking status, use of blood pressure lowering medications, eGFR, FEV1, atrial fibrillation, left ventricular hypertrophy, Q waves and log IL-6.

eGFR, estimated glomerular filtration rate; FEV1, forced expiratory volume in 1 s; IL-6, interleukin-6.

Adding log-NT-pro-BNP to the fully-adjusted models weakened the association between HF and the highest group of the novel frailty score slightly (HR 2.92, 95% CI 1.23 to 6.95, p=0.02); associations between the other scores and incident HF were already non-significant and did not appreciably change from those given in figure 2.

Repeating the first three stepwise main analyses using complete case data only (n=1289) did not materially change the strengths of the associations reported in figure 2.

## Discussion

### Summary

In this cohort of older men, we found an association between frailty—as measured by a novel score combining low gait speed, slow sit-stand time and subjective balance impairment—and incident HF risk, that persisted despite adjustment for comorbidities, known risk factors for HF and biomarkers of inflammation. The Fried frailty phenotype had a non-significant association with increased HF risk in age-adjusted analysis, and this was attenuated further after adjustment for BMI and prevalent MI. By contrast, we found no association between our approximation of the Gill index and incident HF.

The three scores assess different features of frailty. Our novel score incorporated sit-stand time and gait speed, both determined by lower limb muscle strength (itself reflecting sarcopenia^17^), but also sensorimotor, balance and psychological factors.^18 19^ We also included subjective balance difficulties, a complex construct which relates to lower limb strength, peripheral and vestibular sensation,^20^ and central sensorimotor integration and control.^21^ Poor performance in all of these areas was associated with a significantly higher HF risk. This accords with findings that HABC battery score (relating to balance, gait speed and chair-stand) predicted incident HF.^7^

A weaker association was seen between HF risk and the Fried phenotype, which includes gait speed, but also grip strength (dependent on upper-limb muscle power), reduced physical activity, unintentional weight loss and exhaustion (which may reflect reduced energy production, increased energy demand and sarcopenia, but are potentially influenced by comorbidities). Gait speed and balance (as in the novel score) may relate more closely to incident HF than other aspects of the Fried phenotype. Weight loss (‘cardiac cachexia’) and exhaustion are seen in established HF, but may be consequences of the illness, rather than heralds of it.

We found no association between our approximation of the Gill index and incident HF risk, contrasting with prior work.^7^ While the Gill index shares two parameters with our novel score (gait speed and chair-stand, though the scoring of the latter differs), it lacks assessment of balance. Balance (and its underlying determinants) may be more important in determining HF risk. The majority (90%) of men for whom we could calculate this score were identified as non-frail or mildly frail, which may have limited its discriminant ability in this sample. Our calculation of a ‘Gill-like’ score was not identical to the original Gill index, so we cannot refute an association between the Gill index and incident HF. The smaller numbers in our study vs the HABC study^7^ (n=1289 vs n=2825) may also have been under-powered to detect a statistically significant association with the Gill score.

Frail participants may have had asymptomatic, or symptomatic but undiagnosed, HF at baseline. We ran two sensitivity analyses to investigate this possibility. Adjusting for NT-pro-BNP—which is elevated in HF—at baseline made little difference to the association between the novel score and incident HF. In a supplementary analysis, excluding incident HF events within 2 years of baseline, the associations between HF risk and the top group in the novel score were attenuated, but remained increased (although non-statistically significant). A statistically significant association with HF risk was seen in the second-highest group in all analyses. However, because of the small number of cases involved after exclusion, it is difficult to draw firm conclusions from this supplementary analysis. We cannot rule out the possibility of reverse causation.

### Strengths and limitations

Our study has a relatively long and complete follow-up, with multiple baseline assessments, allowing adjustment for many potential confounders. We assessed differential associations between three different measures of frailty status. These frailty scores are straightforward to calculate and easy to replicate in research and clinical practice.

The novel frailty score used in this study was construed as an approximation of the HABC battery score; as both measures are associated with HF risk in two different cohorts, this suggests that they are both testing similar underlying concepts and our novel score may have some external validity. However, our novel frailty score has not been formally validated as a frailty scoring tool, and therefore cannot be used without further validation.

It is possible that residual confounding and reverse causation remains. Although we adjusted for NT-pro-BNP in a sensitivity analysis, we lacked echocardiographic data at the entry examination; some individuals may have had subclinical HF. Our sensitivity analysis excluding HF cases within 2 years suggests that the associations between frailty and HF risk may still have been biased by undiagnosed HF at baseline, though this was based on smaller numbers. Our definition of incident HF relied on physician-diagnosed HF, which may underestimate the true burden of incident HF, although associations between this outcome and HF risk factors reported in our prior work generally accord with prior data^22 23^ suggesting external validity. While we attempted to adjust for the effects of AF, ischaemic heart disease, myocardial damage (as signified by Q waves), stroke and LVH at baseline (all of which are associated with HF risk), our sample size precluded removing these patients from the analyses entirely. ECG determinants of LVH tend to have poor sensitivity (although good specificity)^24^ and therefore more sensitive methods—such as cardiac imaging—may identify more undiagnosed cases of LVH in our sample.

Frailty is strongly associated with all-cause mortality,^25^ which may lead to survivorship bias: frail men may have died earlier from alternative causes and without HF. Frail men from the original cohort may have been less likely to attend the baseline examination and therefore more likely to have been excluded from the analyses. Both of these biases would be expected to weaken associations between incident HF and frailty.

We were unable to determine the HF subtype of those who developed incident HF; HF with reduced ejection fraction and HF with preserved ejection fraction may present with similar clinical features, but are likely to have different underlying pathophysiology^26^ and may show differential associations with frailty.^4^ The ‘novel frailty score’ used here incorporated a subjective measure of balance and may be confounded by multimorbidity. Our cohort was male and almost entirely of White origin, which may limit the generalisability of the findings to women and people of other ethnic groups.

### Implications for future research

Further longitudinal studies should report on the relationship between prevalent frailty and incident HF. Future work should include different frailty measures and should attempt to replicate the strong association seen here between low walk speed, slow chair-stand time, difficulties with balance and incident HF in other cohorts. This association appeared to be independent of known risk factors and inflammation, and so could provide valuable insights into novel mechanisms linking frailty and HF. It could also be developed as a prediction tool for HF risk.

## Data Availability

Data are available on reasonable request. Details on data sharing policy is here: https://www.ucl.ac.uk/epidemiology-health-care/research/primary-care-and-population-health/research/ageing/british-regional-heart-study-brhs-6.
